# Modification of the fatty acid composition in *Arabidopsis* and maize seeds using a stearoyl-acyl carrier protein desaturase-1 (*ZmSAD1*) gene

**DOI:** 10.1186/s12870-016-0827-z

**Published:** 2016-06-14

**Authors:** Hewei Du, Min Huang, Jieyun Hu, Jiansheng Li

**Affiliations:** College of Life Science, Yangtze University, Jingzhou, Hubei 434025 Peoples’s Republic of China; China Agricultural University, National Maize Improvement Center of China, Beijing, 100193 Peoples’s Republic of China; Hubei Collaborative Innovation Center for Grain Industry, Yangtze University, Jingzhou, 434025 Peoples’s Republic of China

**Keywords:** Maize (*Zea mays* L.), *Arabidopsis*, Stearoyl-acyl carrier protein desaturase, Oil engineering, Stearic acid, Oleic oil

## Abstract

**Background:**

Stearoyl-acyl carrier protein desaturase (SAD) is a key enzyme that catalyses the conversion of stearoyl-acyl carrier protein (ACP) to oleoyl-ACP, a precursor for the biosynthesis of polyunsaturated fatty acids. *ZmSAD1* (GenBank: KU949326) is a major QTL for stearic acid content in maize seeds. To investigate the biological function and the application potential of maize *ZmSAD1* in oil biosynthesis, we isolated the full-length *ZmSAD1* cDNA from maize B73 and overexpressed it in *Arabidopsis* and maize.

**Results:**

Under seed-specific overexpression of *ZmSAD1* in *Arabidopsis*, the stearic acid content and the ratio of saturated to unsaturated fatty acids in the seeds were significantly decreased relative to those in the control. Conversely, in transgenic *ZmSAD1* RNAi *Arabidopsis* seeds, the contents of stearic acid and long-chain saturated acids and the ratio of saturated to unsaturated fatty acids were significantly increased; in addition, the oleic acid content was significantly decreased. More importantly, transgenic *ZmSAD1* maize that expressed high levels of *ZmSAD1* in its mature seeds showed reduced stearic acid content (1.57 %) and a lower saturated to unsaturated fatty acid ratio (20.40 %) relative to those (1.64 % and 20.61 %, respectively) of the control. Conversely, down-regulation of *ZmSAD1* in maize resulted in increased levels of stearic acid (1.78 %), long-chain saturated acids (0.85 %) and the ratio of saturated to unsaturated fatty acids (21.54 %) relative to those (1.64 %, 0.74 %, and 20.61 %, respectively) of the control, whereas the oleic acid (32.01 %) level was significantly decreased relative to that (32.68 %) of the control.

**Conclusions:**

Our work demonstrates that the contents of stearic acid, oleic acid, and long-chain saturated acids, and the ratio of saturated to unsaturated fatty acids, are modified in maize seeds by seed-specific overexpression or down-regulation of *ZmSAD1*. Therefore, the *ZmSAD1* gene is a useful tool for engineering the seed oil composition in maize and other crops.

**Electronic supplementary material:**

The online version of this article (doi:10.1186/s12870-016-0827-z) contains supplementary material, which is available to authorized users.

## Background

Triacylglycerides (TAG) is the main form of oil storage in many seeds, composed of fatty acids and glycerol, providing carbon and energy to support the establishment of seedlings after germination [1]. Seed oils are not only a major food source for humans but can also serve as a desirable biofuel alternative to fossil oil worldwide [2, 3]. Many crops, such as sesame, soybean, rapeseed, maize, sunflower, cotton, etc., produce vegetable oils in their seeds. However, the composition and content of fatty acids among these crops vary greatly. The quality and utilisation of seed oils is determined by their fatty acid composition. Saturated fatty acids, such as palmitic (C16:0) and stearic acids (C18:0), are stable and tolerant to heat and oxidation. Certain unsaturated fatty acids, such as oleic (C18:1), linoleic (C18:2), and linolenic (C18:3) acids, are beneficial to human health but susceptible to heat and oxidation [4]. The biosynthesis of fatty acids in plants has been extensively documented, which increases the feasibility of efficiently genetically engineering the composition of fatty acids in seeds.

Fatty acid biosynthesis in higher plants occurs in the chloroplasts of green tissues or in the plastids of non-photosynthetic tissues [5]. In higher plants, the desaturation of long-chain fatty acids is accomplished in sequential steps and begins with the conversion of stearoyl-acyl carrier protein (ACP) to oleoyl-ACP, catalysed by stearoyl-ACP desaturase (SAD, EC 1.12.99.6) [6, 7]. Oleoyl-ACP is a precursor for the biosynthesis of polyunsaturated fatty acids [8]. The SAD enzyme introduces the first double bond into saturated fatty acids and is located on the plastid stroma. Therefore, SAD regulates the amount of unsaturated fatty acids and the ratio of saturated to unsaturated fatty acids in higher plants [9]. Several *SAD* genes from a variety of plants have been isolated and characterised [7, 10–12]. Previous research has shown that the transference of a *SAD* gene into *Brassica napus* and tobacco significantly increases the content of stearic and oleic acids [13, 14]. Maize seeds possess low stearic acid and high oleic acid contents; although oleic acid is beneficial to human health, they are sensitive to oxidation and unstable under high temperature conditions. *SAD* may serve as a useful tool for engineering maize plants that produce a balanced content of stearic and oleic acids in their seeds.

The content and composition of oil in maize is a quantitative trait. Many QTLs associated with the content and composition of oil have been identified and mapped [15–17]. In our previous research, we combined two-dimensional gel electrophoresis (2-DGE) with mass spectrometry (MS) to compare the abundant soluble proteins of early developing embryos in maize. We identified 83 proteins that showed significantly different expression levels between a high-oil (By804) and a regular inbred line (B73), including the ZmSAD protein [18]. The expression level of *ZmSAD* (GenBank: DQ192663) was significantly higher in the By804 seeds than in the B73 seeds [18]. Maize contains two copies of the *ZmSAD* gene, *ZmSAD1* and *ZmSAD2* that were isolated from By804 and B73 and mapped on bin3.05-3.06 and bin8.05-8.06, respectively [19]. The *ZmSAD1* mRNA level was high in the B73 embryos but low in the By804 embryos; conversely, the *ZmSAD2* mRNA level was high in the By804 embryos but low in the B73 embryos. *ZmSAD1* is one of the major QTLs that control stearic acid content [20]. *ZmSAD2* is localized to the oilc8 QTL (phi119-umc1360) and accounts for 7.29 % of the phenotypic variation in kernel oil concentration [17]. Because *ZmSAD1* has a greater effect on oil content, we selected *ZmSAD1* to modify the composition of stearic and oleic acid in maize seeds.

For the genetic engineering of seed oil composition, ideal promoters should show high transcriptional activity and seed specificity. The *Fatty Acid Elongation 1* (*FAE1*) promoter is seed specific [21]. For example, when the *FAE1* promoter was used to control seed-specific overexpression of *ZmSAD2*, the composition of fatty acids in *Arabidopsis* seeds was desirably modified [22]. In this study, we tested the biological function and the potential application of *ZmSAD1* in oil engineering. We transformed *Arabidopsis* and maize with *ZmSAD1* using *Agrobacterium*-mediated transformation and particle bombardment, respectively, and succeeded in modifying the composition of fatty acids in maize seeds. Our results suggest that the *ZmSAD1* gene is a helpful tool for genetically engineering the composition of oil in maize or oil crops.

## Results

### Endogenous *SAD* expression levels are altered in transgenic *Arabidopsis*

**Fig. 1:**
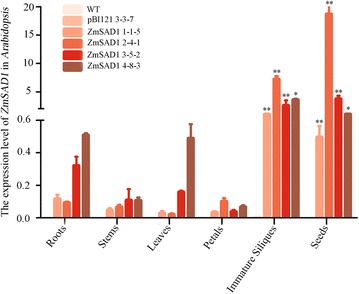
Expression levels of exogenous *ZmSAD1* in various tissues of *Arabidopsis*. All of the experiments were performed with three biological replicates. In each transgenic line, approximately 0.2 g of roots, stems, and leaves were collected from 6 plants. Approximately 0.1 g petals and 0.2 g immature siliques were collected from 8 transgenic plants from each line. Approximately 0.1 g mature seeds collected from 18 transgenic plants were collected from each line. Total RNA samples were isolated from the above tissues. The expression level of *ZmSAD1* was determined by qRT-PCR. The relative transcript levels were calculated using the *Arabidopsis actin1* gene (GenBank: NM179953) as the internal reference. Each bar represents the mean ± SD of three independent biological replicates. Student’s *t*-test was performed. *indicates *p* < 0.05; **indicates *p* < 0.01

We obtained the full-length cDNA of *ZmSAD1* from B73 using the 5’-RACE technique [19]. The *ZmSAD1* overexpression (*ZmSAD1*), antisense *ZmSAD1* (*anti-ZmSAD1*), and *ZmSAD1* RNA interference (*ZmSAD1* RNAi) constructs were generated in the pBI121 vector (Additional file 1: Figure S1), and their expression was under control of the *FAE1* promoter, a seed-specific promoter [21]. The constructs were then used to transform *Arabidopsis*. To assess the expression levels of the exogenous *ZmSAD1* gene in various *Arabidopsis* tissues, qRT-PCR experiments were performed. In the transgenic *ZmSAD1* lines (#1-1-5, 2-4-1, 3-5-2, 4-8-3), exogenous *ZmSAD1* exhibited significantly high expression levels in mature seeds and immature siliques than in roots, stems, leaves, and petals (Fig. 1). Without the presence of *ZmSAD1*, the expression of *ZmSAD1* was undetectable in the WT and transgenic empty vector *Arabidopsis* lines (#3-3-7) (Fig. 1). These results illustrate that the *FAE1* promoter drives high expression levels of *ZmSAD1* in immature siliques and mature seeds.

**Fig. 2:**
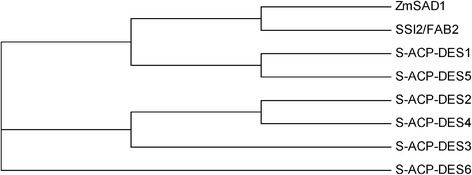
Phylogenetic tree of *ZmSAD1* and seven *Arabidopsis AtSAD* homologous genes. Multiple protein sequence alignment analyses were performed using Clustal X. The phylogenetic tree of the aligned protein sequences was constructed using MEGA 5.0

**Fig. 3:**
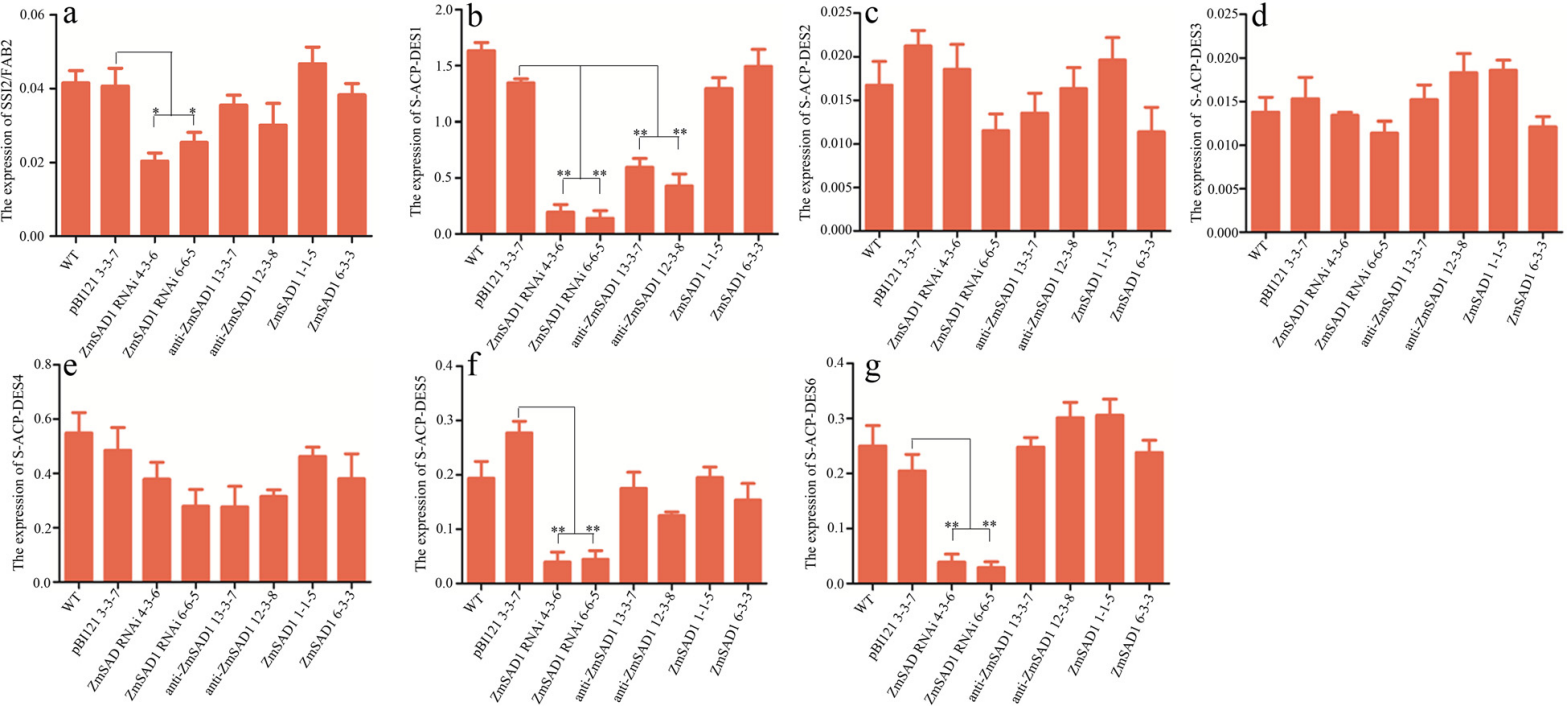
Expression levels of the *Arabidopsis* endogenous *AtSAD* genes in mature seeds. **a**, *SSI2/FAB2*. **b**, *S-ACP-DES1*. **c**, *S-ACP-DES2*. **d**, *S-ACP-DES3*. **e**, *S-ACP-DES4*. **f**, *S-ACP-DES5*. **g**, *S-ACP-DES6*. In each transgenic line, approximately 0.1 g mature seeds collected from 18 plants were used for total RNA isolation. The expression levels of the seven *Arabidopsis* endogenous *AtSAD* were determined by qRT-PCR. Expression levels were calculated using the *Arabidopsis actin1* gene as the internal reference. Each bar represents the mean ± SD of three independent biological replicates. Student’s *t*-test was performed. * indicates p < 0.05; ** indicates p < 0.01

The *Arabidopsis* genome encodes seven homologous *AtSAD* genes: *SSI2/FAB2* (GenBank: At2g43710), *S-ACP-DES1* (stearoyl-acyl carrier protein-desaturase) (GenBank: At5g16240), *S-ACP-DES2* (GenBank: At3g02610), *S-ACP-DES*3 (GenBank: At5g16230), *S-ACP-DES*4 (GenBank: At3g02620), *S-ACP-DES*5 (GenBank: At3g02630), and *S-ACP-DES*6 (GenBank: At1g43800) [23]. An alignment analysis of the deduced amino acid sequences of *ZmSAD1* and the seven *AtSAD* genes was performed using ClustalX. The deduced amino sequences (124 ~ 146 aa, 170 ~ 196 aa, 199 ~ 211 aa, 215 ~ 241 aa, 272 ~ 283 aa) are highly conserved (Additional file 2: Figure S2). A phylogenetic relationship analysis shows that ZmSAD1 shares greater homology with SSI2/FAB2 compared with the other proteins (Fig. 2). To assess the expression level of the *Arabidopsis* endogenous *AtSAD* genes in the mature seeds, a qRT-PCR assay was performed. The results showed that *SSI2/FAB2*, *S-ACP-DES2*, and *S-ACP-DES3* were expressed at lower levels than the other *AtSAD* genes in the mature seeds. In all cases, the expression levels of *S-ACP-DES2*, *S-ACP-DES3* and *S-ACP-DES4* remained unchanged upon introduction of a *ZmSAD1* construct. The expression of *S-ACP-DES1* was significantly decreased in both the transgenic *anti-ZmSAD1* and *ZmSAD1* RNAi plants compared with the empty vector plant (Fig. 3). The expression of *SSI2/FAB2* was slightly decreased in the transgenic *anti-ZmSAD1* seeds compared with the control (transgenic empty vector), although the difference was not statistically significant. The expression levels of *SSI2/FAB2*, *S-ACP-DES5* and *S-ACP-DES6* were significantly decreased in the transgenic *ZmSAD1* RNAi seeds compared with that of control (Fig. 3a, f, g). These results confirm that *ZmSAD1* RNAi and/or *anti-ZmSAD1* suppressed the expression levels of *SSI2/FAB2*, *S-ACP-DES1*, *S-ACP-DES5*, *S-ACP-DES6,* and *SSI2/FAB2* in the transgenic *Arabidopsis* seeds.

### Fatty acid composition and content are modified in transgenic *Arabidopsis* seeds

The compositions of fatty acids in the *Arabidopsis* seeds of transgenic lines were analysed by gas chromatography, together with the transgenic pBI121 empty vector line as the control. The contents of various fatty acids are summarized in Table 1 and Additional file 3: Table S1. With seed-specific *ZmSAD1*, the average content of stearic acid (2.88 %) was significantly decreased compared with control (3.27 %); conversely, with seed-specific silencing using *ZmSAD1* RNAi, the average content of stearic acid (3.62 %) was significantly increased compared with control (Table 1). However, the average contents of oleic acid in the *anti-ZmSAD1* (13.94 %) and *ZmSAD1* RNAi (12.24 %) lines were lower than in the control (15.61 %). The average content of long-chain saturated acids (2.54 %) in the *ZmSAD1* plants was moderately reduced relative to the control (2.84 %), whereas it was significantly increased in the *ZmSAD1* RNAi plants (3.25 %) (Table 1). The ratio of saturated to unsaturated fatty acids was significantly reduced in the *ZmSAD1* plants (17.28 %); conversely, it was significantly increased in both the *anti-ZmSAD1* (18.69 %) and *ZmSAD1* RNAi plants (20.50 %) (Table 1). Therefore, the seed-specific overexpression of the exogenous *ZmSAD1* gene significantly reduced the content of stearic acid and the ratio of saturated to unsaturated fatty acids. The seed-specific down-regulation of endogenous *AtSAD* genes in *Arabidopsis* significantly increased the content of stearic and long-chain saturated fatty acids and the ratio of saturated to unsaturated fatty acids, whereas it significantly decreased the oleic acid content.

**Table 1:** Composition of fatty acids in the *Arabidopsis* seeds harbouring the exogenous *ZmSAD1*, *anti-ZmSAD1*, or *ZmSAD1* RNAi constructs

Lines	Stearic acid		Oleic acid		Long-chain saturated acids (%)		Ratio of saturated to unsaturated fatty acids (%)	
	Content (%)	Mean (%)	Content (%)	Mean (%)	Content (%)	Mean (%)	Content (%)	Mean (%)
pBI121 1-3-5	3.27 ± 0.07	3.27 ± 0.19	15.15 ± 0.29	15.61 ± 0.87	2.98 ± 0.06	2.84 ± 0.21	17.76 ± 0.08	17.71 ± 0.04
pBI121 3-3-7	3.47 ± 0.10		16.62 ± 0.05		2.57 ± 0.07		17.72 ± 0.16	
pBI121 8-5-2	3.08 ± 0.06		15.07 ± 0.43		2.97 ± 0.06		17.69 ± 0.11	
ZmSAD1 1-1-5	2.76 ± 0.11	2.88 ± 0.21**	14.94 ± 0.39	15.21 ± 0.87	2.66 ± 0.11	2.54 ± 0.23	16.77 ± 0.15	17.28 ± 0.56*
ZmSAD1 2-4-1	3.02 ± 0.07		14.04 ± 0.11		2.51 ± 0.21		16.82 ± 0.16	
ZmSAD1 3-5-2	3.12 ± 0.30		15.64 ± 0.23		2.40 ± 0.04		18.02 ± 0.28	
ZmSAD1 4-8-3	2.71 ± 0.06		14.84 ± 0.37		2.63 ± 0.03		17.04 ± 0.27	
ZmSAD1 6-3-3	2.67 ± 0.08		15.15 ± 0.33		2.58 ± 0.13		17.08 ± 0.59	
ZmSAD1 7-2-4	3.01 ± 0.08		16.62 ± 0.46		2.48 ± 0.16		17.97 ± 0.88	
Anti-ZmSAD1 2-8-5	3.26 ± 0.12	3.31 ± 0.15	15.51 ± 0.21	13.94 ± 1.66*	2.46 ± 0.14	2.82 ± 0.40	18.39 ± 0.03	18.69 ± 0.58**
Anti-ZmSAD1 16-5-1	3.45 ± 0.07		15.96 ± 0.23		2.82 ± 0.08		18.01 ± 0.83	
Anti-ZmSAD1 13-3-7	3.49 ± 0.06		13.11 ± 0.07		2.39 ± 0.02		19.47 ± 0.09	
Anti-ZmSAD1 18-6-2	3.15 ± 0.09		12.73 ± 0.28		3.19 ± 0.06		19.09 ± 0.61	
Anti-ZmSAD1 30-2-2	3.20 ± 0.01		12.41 ± 0.26		3.26 ± 0.04		18.51 ± 0.32	
ZmSAD1 RNAi 2-2-7	3.21 ± 0.08	3.62 ± 0.37*	11.77 ± 0.25	12.24 ± 0.53**	3.51 ± 0.08	3.25 ± 0.20**	19.60 ± 0.21	20.50 ± 2.17**
ZmSAD1 RNAi 4-3-6	4.3 ± 0.09		11.58 ± 0.16		3.04 ± 0.11		23.95 ± 0.19	
ZmSAD1 RNAi 6-6-5	3.7 ± 0.05		12.56 ± 0.05		3.37 ± 0.08		21.14 ± 0.18	
ZmSAD1 RNAi 7-4-4	3.20 ± 0.09		12.80 ± 0.24		3.19 ± 0.03		18.33 ± 0.42	
ZmSAD1 RNAi 12-1-5	3.69 ± 0.08		12.50 ± 0.08		3.17 ± 0.12		19.49 ± 0.78	

Student’s *t*-test was performed. *indicates *p* < 0.05; **indicates *p* < 0.01

### Introduction of the *ZmSAD1* gene into maize

**Fig. 4:**
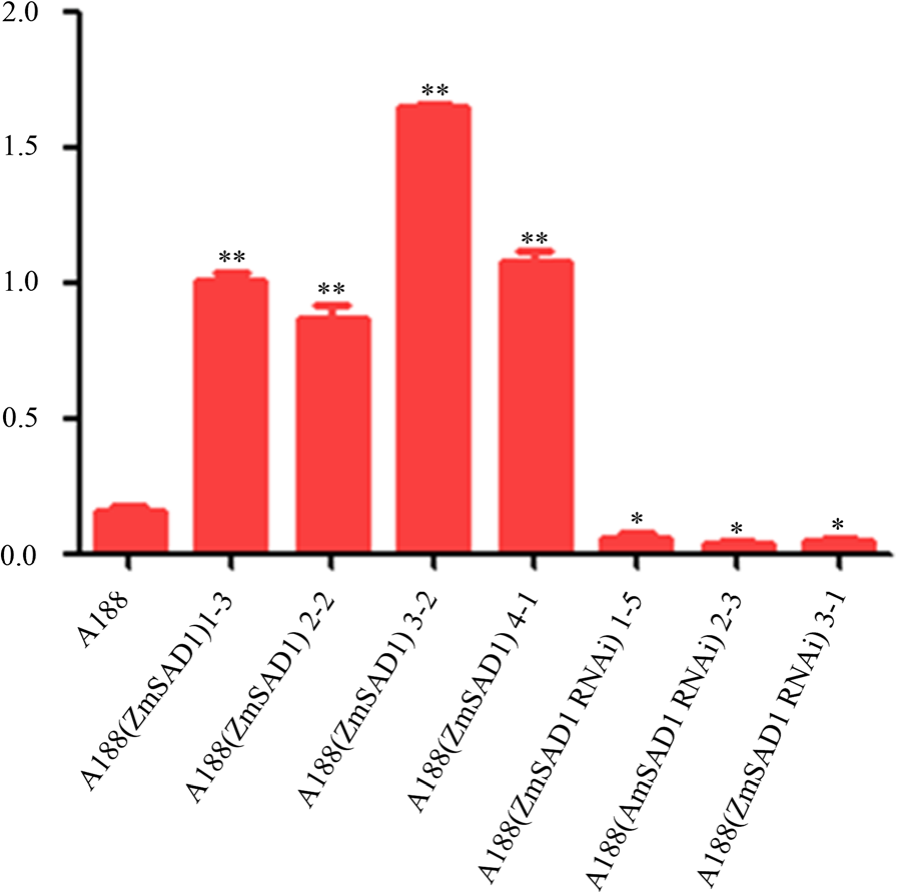
Expression levels of *ZmSAD1* in the mature maize embryos. Ten maize embryos were excised from the mature seeds of A188, A188(ZmSAD1), and A188(ZmSAD1 RNAi). Total RNA samples were isolated from mature embryos. The expression level of *ZmSAD1* was determined for each sample by qRT-PCR. Three biological replicates were performed. The expression levels were calculated using the maize *actin1* gene (MaizeGDB: GRMZM2G126010) as the internal reference. Each bar represents the mean ± SD of three independent biological replicates. Student’s *t*-test was performed. * indicates *p* < 0.05; ** indicates *p* < 0.01

Our results confirmed that the composition of fatty acids in the *Arabidopsis* seeds was modified by the introduction of an exogenous *ZmSAD1* gene and suppressed by *ZmSAD1* RNAi. Because maize oils are one of the major polyunsaturated vegetable oils for human consumption and modification of maize oil composition carries tremendous implications in practical application, we expanded our experiments to maize to modify the composition of fatty acids in maize seeds. We individually transformed the *ZmSAD1* and *ZmSAD1* RNAi constructs under control of the *FAE1* promoter into maize by particle bombardment. Maize calli resistant to bialaphos selection were obtained after particle bombardment (Additional file 4: Figure S3a), and plantlets were regenerated from these calli. The plantlets with well-developed roots were transferred to a growth chamber (Additional file 4: Figure S3b). PCR and Southern blot assays were conducted; the results confirmed that the *ZmSAD1* gene was integrated into the maize genome (Additional file 4: Figure S3c, d). Subsequently, positive *ZmSAD1* and *ZmSAD1* RNAi T_0_ plants were crossed with inbred line A188. The *ZmSAD1* and *ZmSAD1* RNAi constructs were established in the A188 background (sharing approximate 99.4 % similarity) after five back crosses with the recurrent parent A188 followed by one self-pollination. We obtained four A188(ZmSAD1) and three A188(ZmSAD1 RNAi) lines. The *ZmSAD1* expression levels in the maize mature embryos were determined using qRT-PCR. The total *ZmSAD1* expression levels (including endogenous *ZmSAD1* and exogenous *ZmSAD1*) in the A188(ZmSAD1) mature embryos are higher compared with that of the control. In contrast, the *ZmSAD1* expression levels in the A188(ZmSAD1 RNAi) mature seeds are lower than that in the control, indicating that the *ZmSAD1* RNAi construct effectively suppresses expression of the endogenous *ZmSAD1* gene in maize (Fig. 4).

### Overexpression of *ZmSAD1* changes the fatty acid composition in maize seeds

The composition of fatty acids in maize seeds was also analysed using gas chromatography. The contents of various fatty acids are shown in Table 2 and Additional file 5: Table S2. In A188 seeds, the percentage of oleic acid in total fatty acids is 32.68 % (Table 2), which is higher than the percentage of other fatty acids. In the A188(ZmSAD1) mature seeds (#1-5, 2–3, 3–1, 4–5), the average content of stearic acid (1.57 %) and the ratio of saturated to unsaturated acids (20.40 %) are significantly decreased compared with those of the A188 control (1.64 % and 20.61 %, respectively) (Table 2), indicating that seed-specific *ZmSAD1* results in a decrease in the content of stearic and other saturated acids. On the contrary, seed-specific down-regulation of *ZmSAD1* by RNAi increases the average content of stearic acid (1.78 %) and decreases the average content of oleic acid (32.01 %) compared with those of the control (1.64 % and 32.68 %, respectively) (Table 2). The average content of long-chain saturated fatty acids and the ratio of saturated to unsaturated fatty acids are both significantly increased in the A188(*ZmSAD1* RNAi) mature seeds (#1-2, 2–3, 3–3). Conversely, the contents of oleic acid and long-chain saturated acids in the A188(ZmSAD1) mature seeds are not significantly changed compared with those of the control.

**Table 2:** Composition of fatty acids in the maize seeds harbouring *ZmSAD1* or *ZmSAD1* RNAi constructs

Lines	Stearic acid		Oleic acid		Long-chain saturated acids (%)		Ratio of saturated to unsaturated fatty acids (%)	
	Content (%)	Mean (%)	Content (%)	Mean (%)	Content (%)	Mean (%)	Content (%)	Mean (%)
A188	1.64 ± 0.03	1.64 ± 0.03	32.68 ± 0.09	32.68 ± 0.09	0.74 ± 0.01	0.74 ± 0.01	20.61 ± 0.38	20.61 ± 0.38
A188(ZmSAD1) 1-5	1.57 ± 0.02	1.57 ± 0.04*	32.39 ± 1.02	32.85 ± 0.47	0.69 ± 0.04	0.71 ± 0.02	20.48 ± 0.97	20.40 ± 0.19*
A188(ZmSAD1) 2-3	1.52 ± 0.06		32.51 ± 0.53		0.73 ± 0.03		20.63 ± 0.41	
A188(ZmSAD1) 3-1	1.58 ± 0.02		33.11 ± 0.82		0.71 ± 0.02		20.24 ± 0.14	
A188(ZmSAD1) 4-5	1.61 ± 0.05		33.37 ± 1.07		0.71 ± 0.01		20.25 ± 0.26	
A188(ZmSAD1 RNAi) 1-2	1.73 ± 0.03	1.78 ± 0.05*	32.03 ± 0.61	32.01 ± 0.17**	0.86 ± 0.05	0.85 ± 0.04*	21.29 ± 0.37	21.54 ± 0.41**
A188(ZmSAD1 RNAi) 2-3	1.79 ± 0.01		32.17 ± 0.27		0.88 ± 0.03		21.32 ± 0.26	
A188(ZmSAD1 RNAi) 3-3	1.82 ± 0.02		31.83 ± 0.72		0.81 ± 0.06		22.01 ± 0.11	

Student’s *t*-test was performed. *indicates *p* < 0.05; **indicates *p* < 0.01

## Discussion

Reverse and forward genetics are widely used approaches to identify genes of interest. Our group has previously used a proteomics approach by conducting 2-dimensional gel electrophoresis to screen for differentially expressed proteins in the seeds of low and high-oil maize lines. Among the proteins identified in this proteomics study, the ZmSAD protein is expressed at high levels in By804 (a high-oil inbred line) and at low levels in B73 (a regular inbred line) [18]. Therefore, we selected *ZmSAD* as candidate genes to determine their association with the amount and composition of fatty acids. Subsequently, the cDNA sequence of the *ZmSAD* (GenBank: DQ192663) gene was also annotated by an *in silico* approach [18]; the initial in silico gene prediction was further improved. Two copies of the *ZmSAD* gene, *ZmSAD1* and *ZmSAD2*, were isolated by RACE from B73 and By804, respectively, and localized on chromosomes 3 and 8, respectively. The cDNA and deduced amino acid sequences of *ZmSAD1* and *ZmSAD2* are highly homologous [19]. In our previous and current studies, the full-length cDNAs of *ZmSAD1* and *ZmSAD2,* both under control of the *FAE1* promoter, were used to transform *Arabidopsis*. Overexpression of either *ZmSAD1* or *ZmSAD2* leads to a decrease in stearic acid content in *Arabidopsis* seeds (Table 1) [22]. However, whether *ZmSAD1* or *ZmSAD2* is the major gene regulating stearic acid content remains unclear. Later, through mapping, *ZmSAD1* was confirmed to be the major QTL for the content of stearic acid [20]. Therefore, reverse genetics combined with forward genetics proved to be an effective solution in this case.

Stearic acid and oleic acid are regarded as two desirable dietary components of seed oil. Stearic acid has a higher melting point and is more resistant to oxidation compared with unsaturated acids, rendering it suitable for cooking, roasting, and frying [24]. In addition, stearic acid has a neutral effect on total and low-density lipoprotein (LDL) cholesterol levels in the blood [25]. As a monounsaturated fatty acid, oleic acid is beneficial to human health [25]. In this study, we examined the feasibility of modifying the fatty acid composition of maize by elevating or suppressing the expression level of the *ZmSAD1* gene. A188(ZmSAD1 RNAi) seeds have a higher content of stearic acid and long-chain saturated acids and a lower content of oleic acid, whereas A188(ZmSAD1) seeds contain lower levels of stearic acid. However, the modified traits of A188(ZmSAD1), including contents of stearic acid, long-chain saturated acids, and oleic acid, are not the exact opposite to those of A188(ZmSAD1 RNAi). The content of long-chain saturated acids was decreased in A188(ZmSAD1), although the change was not statistically significant.

The contents of stearic acid and oleic acid are approximately 1.64 % and 32.68 %, respectively, in A188 maize; thus oleic acid is about 20 times higher than stearic acid in A188. With the seed-specific overexpression of *ZmSAD1*, the average content of stearic acid in A188(ZmSAD1) was significantly decreased (by 0.07 %) to 1.57 %. The magnitude of this decrease in stearic acid content is small compared with the oleic acid content in A188(ZmSAD1). It also resulted in an insignificant increase in the oleic acid content, insufficient to significantly improve the oleic acid content. Similar results were also found in the *Arabidopsis* transgenic *ZmSAD1* plants. Therefore, we hypothesize that introduction of multiple genes to engineer the fatty acid synthesis pathway is required in order to effectively modify the composition of multiple fatty acids.

The SAD enzyme catalyses the desaturation of stearoyl-ACP to form oleoyl-ACP. ZmSAD1 is highly homologous to ZmSAD2 [19], which indicates a functional similarity between the two genes encoding the proteins. However, *ZmSAD1* is a major QTL for stearic acid, whereas *ZmSAD2* is a QTL for oil content [19]. The difference in the biological functions of *ZmSAD1* and *ZmSAD2* may be associated with their expression levels and their genetic backgrounds. For example, the maize inbred line A188 contains endogenous *ZmSAD1*. With the seed-specific overexpression of *ZmSAD1*, the content of stearic acid and the ratio of saturated to unsaturated fatty acids were successfully modified in A188(ZmSAD1) mature seeds (Table 2), suggesting that the elevated level of *ZmSAD1* is an effective solution for decreasing the stearic acid content in the A188 background. The expression levels of *ZmSAD1* allelic genes vary significantly in different maize genetic backgrounds. Thus, inbred lines with high *ZmSAD1* expression levels may be the best candidates for modifying the fatty acid composition by traditional breeding.

Antisense and RNAi approaches have been used to suppress the expression of endogenous genes and to modify fatty acid compositions [26, 27]. However, the degree of gene silencing under RNAi-mediated suppression is much higher than that under antisense-mediated suppression, particularly in heterozygotes [28]. In the present study, we individually introduced into *Arabidopsis* the anti-*ZmSAD1* and *ZmSAD1* RNAi constructs under control of the same *FAE1* promoter. However, the fatty acid modification in the *Arabidopsis* transgenic anti-*ZmSAD1* seeds was not consistent with that in the *ZmSAD1* RNAi seeds. We hypothesize that this discrepancy may be due to the degree of silencing of endogenous *AtSAD* between anti-*ZmSAD1* and *ZmSAD1* RNAi seeds. Among the 7 *Arabidopsis SAD* genes, *SI2/FAB2*, *S-ACP-DES1*, *S-ACP-DES5*, and *S-ACP-DES6* expression levels were significantly decreased in the transgenic *ZmSAD1* RNAi seeds, whereas only the *S-ACP-DES1* level was decreased in the transgenic anti-*ZmSAD1* seeds, which confirmed that the *ZmSAD1* RNAi construct is more effective than the anti-*ZmSAD1* construct in silencing *AtSAD* genes.

All seven *Arabidopsis* SAD enzymes utilize C18:0-ACP as the preferred substrate [23], which indicates that these proteins can catalyse the desaturation of C18:0-ACP to C18:1-ACP. In *Arabidopsis* transgenic *ZmSAD1* RNAi seeds, the *SSI2/FAB2*, *S-ACP-DES1* and *S-ACP-DES5* levels were significantly decreased, indicating that the C18:0-ACP to C18:1-ACP conversion step in fatty acid synthesis was suppressed. On the contrary, in anti-*ZmSAD1Arabidopsis* seeds, although the *S-ACP-DES1* level was significantly decreased, the *SSI2/FAB2* and *S-ACP-DES5* levels remained unchanged. The unchanged *SSI2/FAB2* and *S-ACP-DES5* levels probably compensated for the decrease of *S-ACP-DES1* in anti-*ZmSAD1* seeds, thereby resulting in no changes in the content of stearic and long-chain saturated fatty acids.

## Conclusions

In this study, *ZmSAD1* and *ZmSAD1* RNAi constructs were transformed into maize; the composition of fatty acids was analysed in mature maize seeds using gas chromatography. With seed-specific overexpression of *ZmSAD1*, the content of stearic acid and the ratio of saturated to unsaturated fatty acids are significantly decreased. Down-regulation of *ZmSAD1* results in increased levels of stearic acid and long-chain saturated acids and increased ratio of saturated to unsaturated fatty acids, whereas the content of oleic acid is significantly decreased. We have demonstrated that the *ZmSAD1* gene is an effective tool for modifying the oil composition of maize or oil crops.

## Methods

### Plant growth

*Arabidopsis* seeds were treated with 70 % ethanol for one minute, soaked in 1 % sodium hypochlorite for 15 min, and rinsed with sterile water 4–5 times. The sterilised seeds were transferred to MS medium and grown in a chamber at 19-21 °C for 10 days. Ten-day old seedlings were planted at a density of 2 plants per 25 cm^2^ in moistened potting soil covered with a nylon window screen. The seedlings were grown in a growth chamber at 19-21 °C with a 16 h/ 8 h light–dark cycle.

Maize plantlets derived from the resistant calli were grown in MS medium for 15 days. The plantlets with well-developed roots were transferred into pots with moistened soil. False-positive transgenic seedlings were excluded by performing a PCR screen. Positive transgenic plants were planted in a greenhouse at 23-25 °C with a 16 h/ 8 h light–dark cycle.

### Binary construct and *Agrobacterium* strain

The *FAE1* (GenBank: AF355982.1) promoter was isolated from *Arabidopsis* genomic DNA by PCR amplification [19, 20], and it was substituted for the CaMV35S promoter in the pBI121 vector. Full-length *ZmSAD1* cDNA derived from B73, the antisense *ZmSAD1* construct (full-length *ZmSAD1* cDNA), and the *ZmSAD1* RNAi construct (part sequence of *ZmSAD1* cDNA) (Additional file 4: Figure S3a, b, c) were cloned into the pBI121 binary vector and the pCAMBIA3301 vector (Additional file 4: Figure S3d, e), which were both under control of the *FAE1* promoter. The pBI121 vector contains a Nos promoter-*NPT II* gene cassette and the pCAMBIA3301 vector contains a CaMV 35S promoter-bar gene cassette for the selection of transformants. Partial maps of the resulting constructs as well as the loop sequence and part of *ZmSAD1* in the RNAi construct are shown in Additional file 1: Figure S1. The pBI121-*ZmSAD*1, pBI121-anti-*ZmSAD1*, pBI121-*ZmSAD1* RNAi, and empty pBI121 vector were mobilized individually into *Agrobacterium* GV3101 by a direct DNA transfer method [29], and their integrity in the *Agrobacterium* cells was confirmed by restriction enzyme analysis after extraction of the plasmid from GV3101.

### *Arabidopsis* and maize transformation

*Arabidopsis* transformation was conducted using the floral dip method according to Clough and Bent [30]. Mature seeds were harvested, dried down, and stored until selection. The seeds were treated with 70 % ethanol for 1 min and 1 % sodium hypochlorite for 15 min and then rinsed five times with sterile water. The sterilized seeds were planted on MS medium with kanamycin (75 μg ml^−1^) and grown in a chamber at 21 °C under a 16 h light (50–100 μ Einsteins m^−2^s^−1^) cycle for 10 days. Kanamycin-resistant seedlings with green leaves and well-established roots were selected from the plates and planted into moistened potting soil.

Maize calli derived from immature Hi-II zygotic embryos were used as the target tissues for bombardment. The bombardment and subsequent selection of bialaphos-resistant calli were performed according to Frame et al. [31]. Briefly, the calli were transferred to osmotic medium and then bombarded two times. A total of 40 petri dishes of calli were chosen for bombardment with the *ZmSAD1* or *ZmSAD1* RNAi construct. The bombarded calli were incubated at 28 °C in the dark. After selection and regeneration, the plantlets grew out from the bar-resistant calli. Genomic DNA was isolated from the leaves of the putative transgenic maize plantlets, and PCR and Southern blot assays were performed to confirm the presence of transgenes. Twenty micrograms of each DNA sample was digested with EcoRI at 37 °C overnight. The digestion products were fractionated on a 0.8 % agarose gel and then transferred to a nylon membrane. The *FAE1* promoter probe was labelled with DIG using the PCR method. A Southern blot analysis was conducted according to the protocol of the Roche Southern Blot Kit (Roche Applied Science, Mannheim, Germany). Well-developed positive T_0_ transgenic *ZmSAD1* and *ZmSAD1* RNAi plants were used as the donor to cross with maize inbred line A188, which was used as the recurrent parent. After five backcrosses and one self-pollination coupled with molecular marker-assisted selection for the transgene, the *ZmSAD1* and *ZmSAD1* RNAi constructs were introduced into the A188 genetic background.

### *ZmSAD1* expression

All of the experiments were performed with three biological replicates. Ninety-six well-developed seedlings from each homozygous transgenic T_3_ line of *Arabidopsis* were planted in a growth chamber. After four weeks, approximately 0.2 g of the roots, leaves, and stems were collected from 6 plants. At the flowering stage, approximately 0.1 g of the petals and 0.2 g of the immature siliques were collected from 8 plants. The seeds from each homozygous line were collected from 18 plants and pooled. Approximately 0.1 g of the mature seeds were used for further RNA isolation. In maize, 10 mature embryos from each line were excised from the transgenic or A188 seeds and used for RNA isolation.

Total RNA was isolated from various *Arabidopsis* tissues or maize mature embryos using TRIzol reagent (Invitrogen, Carlsbad, CA, USA). First-strand cDNA was synthesised using SuperScript-II reverse transcriptase according to the manufacturer’s instructions (Invitrogen). The *actin1* genes of *Arabidopsis* and maize (GenBank: NM179953 and MaizeGDB: GRMZM2G126010) were used as the endogenous controls to normalize the expression data (Additional file 6: Table S3). Real-time PCR was conducted using the SYBR Real-time PCR kit (Takara Japan) with IQTM SYBR® Green Supermixture according to the manufacturer’s instructions (Bio-Rad USA). The reaction condition was as follows: 94 °C for 1 min, followed by 40 cycles of 95 °C for 10 s, 55 °C for 10 s, and 72 °C for 15 s.

### Determination of fatty acid composition and content

*Arabidopsis* seeds were harvested from homozygous T_3_ transgenic plants. Mature seeds from each transgenic line were collected from 18 plants, pooled, and subjected to a fatty acid analysis. Ten maize seeds from each line were subjected to a lipid analysis. Seeds of the transgenic pBI121 empty vector in *Arabidopsis* and maize inbred line A188 were used as the controls. Approximately 0.1 g of ground seeds of *Arabidopsis* and 0.2 g of ground seeds of maize were used for each assay. All of the experiments were performed with three biological repeats. The total lipid was extracted from the ground seeds and content analysed as described by Sukhija and Palmquist [32]; the fatty acid composition was analysed using gas chromatography (Hewlett-Packard, Palo Alto, CA, USA). In the present study, the long-chain saturated acids include C20:0, C22:0, and C24:0; the saturated fatty acids include C14:0, C16:0, C18:0, C20:0, C22:0, and C24:0; the unsaturated fatty acids include C16:1, C18:1, C18:2, and C18:3. Fatty acids that are present at low levels, including C10:0, C12:0, were not considered in this study. The content of each fatty acid was expressed as the percentage of total fatty acids.

### Sequence alignment and phylogenetic tree construction

Alignment analyses of ZmSAD1 and the seven AtSAD protein sequences were performed using ClustalX [33]. Phylogenetic trees of the aligned ZmSAD1 and AtSAD protein sequences were constructed using MEGA version 5.0 [34] via the neighbour-joining (NJ) method with the following parameters: Poisson correction, pairwise deletion, and bootstrapping (1000 replicates; random seeds).

## Abbreviations

2-DGE, two-dimensional gel electrophoresis; ACP, acyl carrier protein; CaMV35S, CaMV35S promoter; CDS, coding sequence; DIG, digoxin; FAE1, Fatty Acid Elongation 1; MS, mass spectrometry; qRT-PCR, quantitative real time RT-PCR; RACE, rapid-amplification of cDNA ends; SAD and S-ACP-DES, Stearoyl-acyl carrier protein desaturase; TAG, triacylglycerides.

## Additional files

Additional file 1: Figure S1. Schematic diagrams of the different *ZmSAD1* constructs. (a, d) *ZmSAD1* constructs; (b) *anti-ZmSAD1*; and (c, e) *ZmSAD1* RNAi (not to scale). a, b, and c were in the pBI121 vector and used for *Arabidopsis* transformation. d and e were in the pCAMBIA3301 vector and used for maize transformation. (f) Part of the *ZmSAD1* cDNA sequence used for RNAi. (g) Sequence used as a loop in the RNAi construct. **LB** and **RB**, T-DNA left and right borders, respectively; **Pnos**, nopaline synthase gene promoter; **Tnos**, nopaline synthase gene terminator; **NPTII**, neomycin phosphotransferase II; ***FAE1***, promoter of fatty acid elongation 1 condensing enzyme; ***SAD1***, stearoyl-acyl carrier protein desaturase1; **bar**, phosphinothricin acetyltransferase; **T**35s, CaMV 35S terminator; and P35s: CaMV35S promoter. The full-length cDNA of *ZmSAD1* was used in the *ZmSAD1* and *anti-ZmSAD1* constructs. (DOCX 964 kb) Additional file 2: Figure S2. Protein sequence alignment of *ZmSAD1* and seven *Arabidopsis AtSAD* genes. Multiple protein sequence alignment analyses were performed using Clustal X. GenBank: At2g43710 (*SSI2/FAB2*), At5g16240 (*S-ACP-DES1*), At3g02610 (*S-ACP-DES2*), At5g16230 (*S-ACP-DES*3), At3g02620 (*S-ACP-DES*4), At3g02630 (*S-ACP-DES*5), At1g43800 (*S-ACP-DES*6), and *ZmSAD1* (GenBank: KU949326). Positions are indicated relative to the *ZmSAD1* protein sequence. (TIF 2280 kb) Additional file 3: Table S1. Composition of fatty acids in the transgenic *ZmSAD1 Arabidopsis* mature seeds (DOCX 17 kb) Additional file 4: Figure S3. Generation and confirmation of transgenic *ZmSAD1* and *ZmSAD1* RNAi maize plants. a, Resistant calli (indicated by arrows). b, Regenerated plantlets grown in a growth chamber. c, Detection of the transgene. Lane 1, DL2000 DNA marker. Lane 2, positive control (pCAMBIA3301-*FAE1*-*ZmSAD1* plasmid). Lane 3, negative control (maize inbred line A188). Lane 4–13, transgenic *ZmSAD1* plants. Lane 14, null. Lane 15–24, transgenic *ZmSAD1* RNAi plants. Genomic DNA was isolated from the leaves of T_0_ transgenic plants and used in the PCR and Southern blot analyses. d, Southern blot analysis. Lane 1, λDNA/HindIII marker. Lane 2, pCAMBIA3301-FAE1-*ZmSAD1* digested with EcoRI. Lane 3, non-transgenic plant (maize inbred line A188). Lane 4–5, positive transgenic plants. The *FAE1* fragment was labelled with DIG via PCR amplification and used as probe in the Southern blot analysis. (TIF 5092 kb) Additional file 5: Table S2. Composition of fatty acids in the transgenic *ZmSAD1* maize seeds. (DOCX 15 kb) Additional file 6: Table S3. Primers used in this study. (DOCX 14 kb)
