# Supplementary material for: Modification of the fatty acid composition in Arabidopsis and maize seeds using a stearoyl-acyl carrier protein desaturase-1 (ZmSAD1) gene
Source: BMC Plant Biol. 2016 Jun 14;16:137. doi: 10.1186/s12870-016-0827-z (PMC4906915; doi:10.1186/s12870-016-0827-z)
Supplement: Additional file 1: Figure S1. — Schematic diagrams of the different ZmSAD1 constructs. (a, d) ZmSAD1 constructs; (b) anti-ZmSAD1; and (c, e) ZmSAD1 RNAi (not to scale). a, b, and c were in the pBI121 vector and used for Arabidopsis transformation. d and e were in the pCAMBIA3301 vector and used for maize transformation. (f) Part of the ZmSAD1 cDNA sequence used for RNAi. (g) Sequence used as a loop in the RNAi construct. LB and RB, T-DNA left and right borders, respectively; Pnos, nopaline synthase gene promoter; Tnos, nopaline synthase gene terminator; NPTII, neomycin phosphotransferase II; FAE1, promoter of fatty acid elongation 1 condensing enzyme; SAD1, stearoyl-acyl carrier protein desaturase1; bar, phosphinothricin acetyltransferase; T35s, CaMV 35S terminator; and P35s: CaMV35S promoter. The full-length cDNA of ZmSAD1 was used in the ZmSAD1 and anti-ZmSAD1 constructs. (DOCX 964 kb) [file 12870_2016_827_MOESM1_ESM.docx]

**Additional file 1: Figure S1** Schematic diagrams of the different *ZmSAD1* constructs. **a and** **d**, *ZmSAD1*constructs. **b**, *anti-ZmSAD1*. **c and** **e**, *ZmSAD1* RNAi (not to scale). **f**, the loop sequence in RNAi-mediated construct. **g**, the part sequence of *ZmSAD1* in RNAi-mediated construct. **a**, **b**, and **c** are in the pBI121 vector and used for *Arabidopsis* transformation. **d** and **e** are in the pCAMBIA3301 vector and used for maize transformation. **LB** and **RB**, T-DNA left and right borders, respectively; **Pnos**, nopaline synthase gene promoter; **Tnos**, nopaline synthase gene terminator; **NPTII**, neomycin phosphotransferase II; ***FAE1***, promoter of fatty acid elongation 1 condensing enzyme; *Zm****SAD1***, stearoyl-acyl carrier protein desaturase1; **bar**, phosphinothricin acetyltransferase; **T**35s, CaMV 35S terminator; P35s: CaMV35S promoter. The full-length cDNA of *zmSAD1* was use as *ZmSAD1* and *anti-ZmSAD1* constructs, respectively.


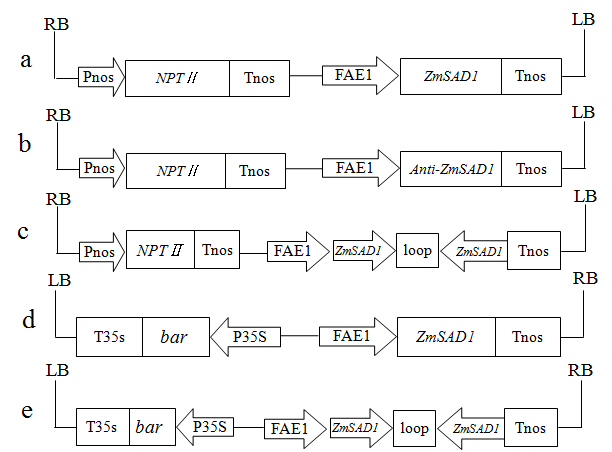


f. The loop sequence

CCTGCAGGTCTAGTTTTTCTCCTTCATTTTCTTGGTTAGGACCCTTTTCTCTTTTTATTTTTTTGAGCTTTGATCTTTCTTTAAACTGATCTATTTTTTAATTGATTGGTTATGGTGTAAATATTACATAGCTTTAACTGATAATCTGATTACTTTATTTCGTGTGTCTATGATGATGATGATAGTTACAGAGCCCGGGC

g. The *ZmSAD1* cDNA fragment in RNAi-mediated construct

TTGGTGGGAGACATGATTACCGAGGAAGCTCTACCAACATACCAGACTATGCTTAACACCCTCGATGGTGTCAGAGATGAGACAGGTGCAAGCCCCACTGCCTGGGCTGTTTGGACGAGGGCATGGACTGCTGAGGAGAACAGGCATGGTGATCTGCTCAACAAGTATATGTACCTCACTGGGAGGGTGGATATGAGGCAGATTGAGAAGACAATTCAGTATCTTATTGGCTCTGGAATGGATCCTAGGACTGAGAATAATCCTTATCTTGGTTTCATCTACACCTCCTTCCAAGAGCGGGCGACCTTCATCTCACACGGGAACACTGCT
