# Supplementary figures and images for: Modification of the fatty acid composition in Arabidopsis and maize seeds using a stearoyl-acyl carrier protein desaturase-1 (ZmSAD1) gene
Source: BMC Plant Biol. 2016 Jun 14;16:137. doi: 10.1186/s12870-016-0827-z (PMC4906915; doi:10.1186/s12870-016-0827-z)

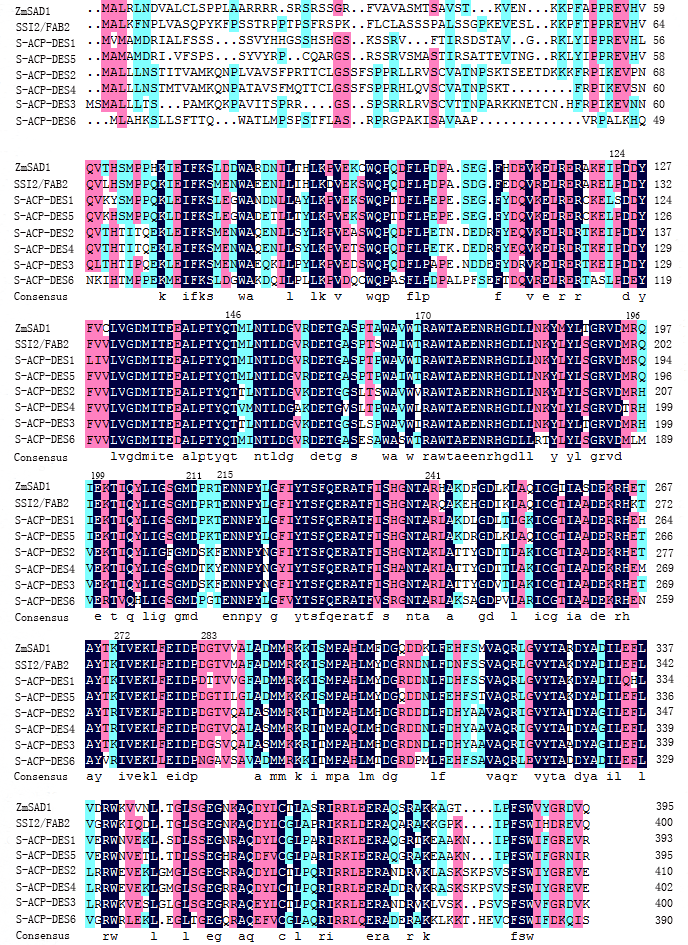

Supplement: Additional file 2: Figure S2. — Protein sequence alignment of ZmSAD1 and seven Arabidopsis AtSAD genes. Multiple protein sequence alignment analyses were performed using Clustal X. GenBank: At2g43710 (SSI2/FAB2), At5g16240 (S-ACP-DES1), At3g02610 (S-ACP-DES2), At5g16230 (S-ACP-DES3), At3g02620 (S-ACP-DES4), At3g02630 (S-ACP-DES5), At1g43800 (S-ACP-DES6), and ZmSAD1 (GenBank: KU949326). Positions are indicated relative to the ZmSAD1 protein sequence. (TIF 2280 kb) [file 12870_2016_827_MOESM2_ESM.tif]

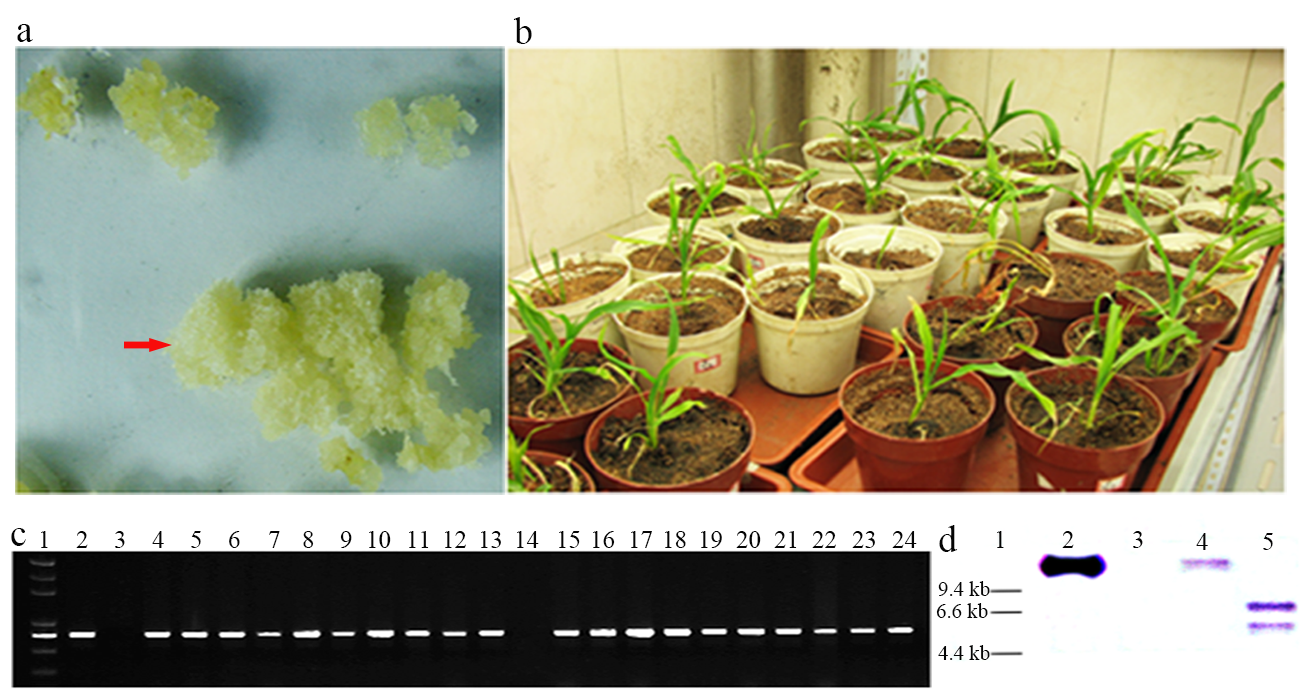

Supplement: Additional file 4: Figure S3. — Generation and confirmation of transgenic ZmSAD1 and ZmSAD1 RNAi maize plants. a, Resistant calli (indicated by arrows). b, Regenerated plantlets grown in a growth chamber. c, Detection of the transgene. Lane 1, DL2000 DNA marker. Lane 2, positive control (pCAMBIA3301-FAE1-ZmSAD1 plasmid). Lane 3, negative control (maize inbred line A188). Lane 4–13, transgenic ZmSAD1 plants. Lane 14, null. Lane 15–24, transgenic ZmSAD1 RNAi plants. Genomic DNA was isolated from the leaves of T0 transgenic plants and used in the PCR and Southern blot analyses. d, Southern blot analysis. Lane 1, λDNA/HindIII marker. Lane 2, pCAMBIA3301-FAE1-ZmSAD1 digested with EcoRI. Lane 3, non-transgenic plant (maize inbred line A188). Lane 4–5, positive transgenic plants. The FAE1 fragment was labelled with DIG via PCR amplification and used as probe in the Southern blot analysis. (TIF 5092 kb) [file 12870_2016_827_MOESM4_ESM.tif]
