# Supplementary material for: Modification of the fatty acid composition in Arabidopsis and maize seeds using a stearoyl-acyl carrier protein desaturase-1 (ZmSAD1) gene
Source: BMC Plant Biol. 2016 Jun 14;16:137. doi: 10.1186/s12870-016-0827-z (PMC4906915; doi:10.1186/s12870-016-0827-z)
Supplement: Additional file 3: Table S1. — Composition of fatty acids in the transgenic ZmSAD1 Arabidopsis mature seeds (DOCX 17 kb) [file 12870_2016_827_MOESM3_ESM.docx]

| Lines | C14:0（%） | C16:0（%） | C20:0（%） | C22:0（%） | C24:0（%） | C16:1（%） | C18:2（%） | C18:3（%） | C20:1（%） | saturated fatty acids (%) | unsaturated fatty acids (%) |
| --- | --- | --- | --- | --- | --- | --- | --- | --- | --- | --- | --- |
|  |  |  |  |  |  |  |  |  |  |  |  |
| pBI121 1-3-5 | 0.29±0.02 | 8.54±0.13 | 2.28±0.02 | 0.41±0.01 | 0.29±0.01 | 0.38±0.03 | 30.56±0.67 | 17.86±0.23 | 20.97±0.82 | 15.08±0.25 | 84.92±0.25 |
| pBI121 3-3-7 | 0.30±0.01 | 8.71±0.09 | 1.85±0.01 | 0.40±0.03 | 0.32±0.03 | 0.40±0.01 | 30.31±0.93 | 18.24±0.31 | 19.38±0.47 | 15.05±0.43 | 84.95±0.43 |
| pBI121 8-5-2 | 0.31±0.04 | 8.67±0.11 | 2.26±0.04 | 0.42±0.02 | 0.29±0.01 | 0.40±0.01 | 30.73±1.02 | 18.65±0.47 | 20.12±0.53 | 15.03±0.21 | 84.97±0.21 |
| ZmSAD1 1-1-5 | 0.43±0.03 | 8.51±0.08 | 1.91±0.02 | 0.42±0.03 | 0.33±0.02 | 0.66±0.05 | 30.56±.042 | 19.56±0.71 | 19.92±0.37 | 14.36±0.92 | 85.64±0.92 |
| ZmSAD1 2-4-1 | 0.38±0.05 | 8.49±0.14 | 1.50±0.03 | 0.63±0.07 | 0.38±0.04 | 0.58±0.04 | 30.64±0.83 | 19.67±0.87 | 20.66±0.09 | 14.40±0.68 | 85.59±0.68 |
| ZmSAD1 3-5-2 | 0.43±0.02 | 9.32±0.09 | 1.71±0.01 | 0.33±0.02 | 0.36±0.05 | 0.43±0.02 | 31.23±0.16 | 17.66±0.54 | 19.76±0.71 | 15.27±1.03 | 84.72±1.03 |
| ZmSAD1 4-8-3 | 0.45±0.02 | 8.77±0.03 | 1.99±0.06 | 0.41±0.01 | 0.23±0.01 | 0.55±0.03 | 30.92±0.53 | 19.76±0.93 | 19.37±0.46 | 14.56±0.46 | 85.44±0.46 |
| ZmSAD1 6-3-3 | 0.47±0.07 | 8.87±0.07 | 1.85±0.04 | 0.46±0.04 | 0.27±0.01 | 0.37±0.02 | 30.61±0.47 | 19.19±1.09 | 20.08±0.31 | 14.59±0.38 | 85.40±0.38 |
| ZmSAD1 7-2-4 | 0.46±0.04 | 9.28±0.03 | 1.87±0.09 | 0.33±0.02 | 0.28±0.02 | 0.43±0.03 | 30.57±0.33 | 18.74±0.93 | 18.41±0.07 | 15.23±0.27 | 84.77±0.27 |
| Anti-ZmSAD1 2-8-5 | 0.50±0.03 | 9.31±0.02 | 1.67±0.03 | 0.40±0.01 | 0.39±0.02 | 0.50±0.04 | 30.31±0.92 | 18.05±0.32 | 20.10±0.22 | 15.53±0.89 | 84.47±0.89 |
| Anti-ZmSAD1 16-5-1 | 0.39±0.03 | 8.60±0.06 | 2.07±0.05 | 0.36±0.04 | 0.39±0.03 | 0.48±0.02 | 30.71±1.10 | 18.64±0.08 | 18.95±0.58 | 15.26±0.77 | 84.74±0.77 |
| Anti-ZmSAD1 13-3-7 | 0.33±0.01 | 10.09±0.10 | 1.72±0.03 | 0.32±0.02 | 0.35±0.05 | 0.59±0.04 | 30.76±0.78 | 18.70±0.11 | 20.54±0.37 | 16.30±0.59 | 83.70±0.59 |
| Anti-ZmSAD1 18-6-2 | 0.30±0.03 | 9.39±0.07 | 2.26±0.08 | 0.47±0.05 | 0.46±0.07 | 0.50±0.01 | 30.63±0.83 | 19.86±0.25 | 20.25±0.47 | 16.03±1.13 | 83.97±1.13 |
| Anti-ZmSAD1 30-2-2 | 0.39±0.04 | 8.77±0.04 | 2.35±0.04 | 0.49±0.02 | 0.42±0.03 | 0.70±0.06 | 30.87±0.31 | 19.37±0.31 | 21.03±0.91 | 15.62±0.43 | 84.38±0.43 |
| ZmSAD1 RNAi 2-2-7 | 0.36±0.01 | 9.31±0.11 | 2.51±0.03 | 0.58±0.04 | 0.42±0.02 | 0.66±0.05 | 30.61±0.61 | 19.37±0.33 | 21.20±0.43 | 16.39±0.52 | 83.61±0.52 |
| ZmSAD1 RNAi 4-3-6 | 0.55±0.07 | 11.43±0.13 | 2.41±0.06 | 0.31±0.01 | 0.32±0.01 | 0.57±0.03 | 30.39±0.83 | 17.95±0.09 | 20.19±0.37 | 19.32±0.76 | 80.68±0.76 |
| ZmSAD1 RNAi 6-6-5 | 0.40±0.02 | 9.98±0.07 | 2.44±0.02 | 0.54±0.03 | 0.39±0.03 | 0.52±0.03 | 30.85±0.47 | 18.27±0.27 | 20.35±0.44 | 17.45±0.29 | 82.55±0.29 |
| ZmSAD1 RNAi 7-4-4 | 0.31±0.01 | 8.79±0.02 | 2.29±0.01 | 0.46±0.02 | 0.44±0.07 | 0.48±0.01 | 30.57±0.52 | 19.20±0.61 | 21.46±0.30 | 15.49±0.43 | 84.51±0.43 |
| ZmSAD1 RNAi 12-1-5 | 0.38±0.03 | 9.07±0.08 | 2.38±0.05 | 0.43±0.05 | 0.36±0.03 | 0.52±0.04 | 30.64±0.81 | 19.04±0.72 | 20.99±0.07 | 16.31±0.25 | 83.69±0.25 |

**Additional file 3:Table S1** Composition of fatty acids in the transgenic *ZmSAD1Arabidopsis* mature seeds
