# Supplementary material for: Modification of the fatty acid composition in Arabidopsis and maize seeds using a stearoyl-acyl carrier protein desaturase-1 (ZmSAD1) gene
Source: BMC Plant Biol. 2016 Jun 14;16:137. doi: 10.1186/s12870-016-0827-z (PMC4906915; doi:10.1186/s12870-016-0827-z)
Supplement: Additional file 5: Table S2. — Composition of fatty acids in the transgenic ZmSAD1 maize seeds. (DOCX 15 kb) [file 12870_2016_827_MOESM5_ESM.docx]

| Lines | C14:0（%） | C16:0（%） | C20:0（%） | C22:0（%） | C24:0（%） | C16:1（%） | C18:2（%） | C18:3（%） | C20:1（%） | saturated fatty acids (%) | unsaturated fatty acids (%) |
| --- | --- | --- | --- | --- | --- | --- | --- | --- | --- | --- | --- |
| A188 | 0.03±0.01 | 14.60±0.48 | 0.41±0.02 | 0.12±0.02 | 0.21±0.01 | 0.13±0.03 | 49.08±1.29 | 0.78±0.03 | 0.32±0.03 | 17.01±0.83 | 82.99±0.83 |
| A188(ZmSAD1) 1-3 | 0.03±0.01 | 14.71±0.25 | 0.37±0.01 | 0.14±0.01 | 0.18±0.02 | 0.16±0.02 | 49.23±1.32 | 0.82±0.05 | 0.39±0.05 | 17.00±0.32 | 82.99±0.32 |
| A188(ZmSAD1) 2-2 | 0.04±0.01 | 14.81±0.19 | 0.36±0.02 | 0.16±0.02 | 0.21±0.01 | 0.14±0.01 | 49.12±0.74 | 0.76±0.02 | 0.37±0.03 | 17.10±0.73 | 82.90±0.73 |
| A188(ZmSAD1) 3-2 | 0.03±0.01 | 14.51±0.91 | 0.43±0.04 | 0.12±0.02 | 0.16±0.03 | 0.13±0.01 | 48.85±0.38 | 0.70±0.03 | 0.38±0.06 | 16.83±0.26 | 83.17±0.26 |
| A188(ZmSAD1) 4-1 | 0.03±0.01 | 14.49±0.74 | 0.39±0.03 | 0.15±0.03 | 0.17±0.02 | 0.13±0.02 | 48.61±1.03 | 0.71±0.04 | 0.34±0.02 | 16.84±0.11 | 83.16±0.11 |
| A188(ZmSAD1 RNAi) 1-5 | 0.05±0.02 | 14.91±0.32 | 0.46±0.02 | 0.16±0.02 | 0.24±0.04 | 0.16±0.03 | 48.95±0.91 | 0.95±0.12 | 0.35±0.01 | 17.55±0.23 | 82.44±0.23 |
| A188(ZmSAD1 RNAi) 2-3 | 0.03±0.01 | 14.87±0.94 | 0.51±0.04 | 0.14±0.02 | 0.23±0.03 | 0.15±0.02 | 48.89±0.57 | 0.86±0.03 | 0.36±0.02 | 17.57±0.52 | 82.43±0.52 |
| A188(ZmSAD1 RNAi) 3-1 | 0.04±0.01 | 15.37±1.03 | 0.44±0.01 | 0.19±0.04 | 0.18±0.02 | 0.15±0.03 | 48.80±1.09 | 0.80±0.06 | 0.38±0.05 | 18.04±0.37 | 81.96±0.37 |

**Additional file 5: Table S2** Composition of fatty acids in the transgenic *ZmSAD1*maize seeds
