# Supplementary material for: Modification of the fatty acid composition in Arabidopsis and maize seeds using a stearoyl-acyl carrier protein desaturase-1 (ZmSAD1) gene
Source: BMC Plant Biol. 2016 Jun 14;16:137. doi: 10.1186/s12870-016-0827-z (PMC4906915; doi:10.1186/s12870-016-0827-z)
Supplement: Additional file 6: Table S3. — Primers used in this study. (DOCX 14 kb) [file 12870_2016_827_MOESM6_ESM.docx]

**Additional file 6: Table S3** Primers used in this study

| Primers | Sequence (5´-3´) | Usage |
| --- | --- | --- |
| FAE1 Forward | GAGTGGTTCTTGAGACCGATGA | Amplifying or labelling FAE1 promoter by PCR |
| FAE1 Reverse | ATGGAGGAAGCTATGAGATGTATT | Amplifying or labelling FAE1 promoter by PCR |
| ZmSAD1 RNAi Forward | TTGGTGGGAGACATGATTACC | Amplifying *ZmSAD1* fragment for RNAi-mediated construct |
| ZmSAD1 RNAi Reverse | AGCAGTGTTCCCGTGTGAGAT | Amplifying *ZmSAD1* fragment for RNAi-mediated construct |
| SSI2/FAB2 qForward | AGTTAGGGATGAAACAGGTGC | qRT-PCR for *SSI2/FAB2* gene |
| SSI2/FAB2 qReverse | ATAGATGAAGCCAAGGTAGGG | qRT-PCR for *SSI2/FAB2* gene |
| S-ACP-DES1 qForward | TTGATGTATGATGGTCGTGATG | qRT-PCR for *S-ACP-DES1* gene |
| S-ACP-DES1 qReverse | TGAATGGTATGTTTTTTGCTGC | qRT-PCR for *S-ACP-DES1 g*ene |
| S-ACP-DES2 qForward | AACGGTGGCTATGAAGCAGA | qRT-PCR for *S-ACP-DES2* gene |
| S-ACP-DES2 qReverse | TGTGGGTCACTTGGTTTGGT | qRT-PCR for *S-ACP-DES2* gene |
| S-ACP-DES3 qForward | ATCATTCCAAGAGCGAGCAA | qRT-PCR for *S-ACP-DES3* gene |
| S-ACP-DES3 qReverse | ATCTTCGTGTACGCCGTCTC | qRT-PCR for *S-ACP-DES3* gene |
| S-ACP-DES4 qForward | TTTTATCTCCCACGCCAACA | qRT-PCR for *S-ACP-DES4* gene |
| S-ACP-DES4 qReverse | TTCGCTTCCTCATCATACTCG | qRT-PCR for *S-ACP-DES4* gene |
| S-ACP-DES5 qForward | GCTTCTACGATTCGCTCTGC | qRT-PCR for *S-ACP-DES5* gene |
| S-ACP-DES5 qReverse | GTAGGCTGCCACGATTTCTC | qRT-PCR for *S-ACP-DES5* gene |
| S-ACP-DES6 qForward | GCAACCCGCTTCTTTCTTAC | qRT-PCR for *S-ACP-DES6* gene |
| S-ACP-DES6 qReverse | CTCGTCCCTTACGCCATCAA | qRT-PCR for *S-ACP-DES6* gene |
| ZmSAD1 qForward | AGGCTTGGCGTTTACACT | qRT-PCR for maize *ZmSAD1* gene |
| ZmSAD1 qReverse | AGCAGGTCTTGTCTAAGCAGT | qRT-PCR for maize *ZmSAD1* gene |
| Ara-Actin1 qForward | CCCCTGCTATGTATGTGGCTAT | qRT-PCR for *Arabidopsis* *Actin1* gene |
| Ara-Actin1 qReverse | GACAATTTCACGCTCTGCTGT | qRT-PCR for *Arabidopsis* *Actin1* gene |
| Maize-Actin1 qForward | TGTTGCTATCCAGGCTGTTCT | qRT-PCR for maize *Actin1* gene |
| Maize-Actin1 qReverse | TCATTAGGTGGTCGGTGAGGT | qRT-PCR for maize *Actin1* gene |
